# Hodge Decomposition of Information Flow on Small-World Networks

**DOI:** 10.3389/fncir.2016.00077

**Published:** 2016-09-28

**Authors:** Taichi Haruna, Yuuya Fujiki

**Affiliations:** Department of Planetology, Graduate School of Science, Kobe UniversityKobe, Japan

**Keywords:** small-world network, random threshold network, transfer entropy, Hodge decomposition, functional brain networks

## Abstract

We investigate the influence of the small-world topology on the composition of information flow on networks. By appealing to the combinatorial Hodge theory, we decompose information flow generated by random threshold networks on the Watts-Strogatz model into three components: gradient, harmonic and curl flows. The harmonic and curl flows represent globally circular and locally circular components, respectively. The Watts-Strogatz model bridges the two extreme network topologies, a lattice network and a random network, by a single parameter that is the probability of random rewiring. The small-world topology is realized within a certain range between them. By numerical simulation we found that as networks become more random the ratio of harmonic flow to the total magnitude of information flow increases whereas the ratio of curl flow decreases. Furthermore, both quantities are significantly enhanced from the level when only network structure is considered for the network close to a random network and a lattice network, respectively. Finally, the sum of these two ratios takes its maximum value within the small-world region. These findings suggest that the dynamical information counterpart of global integration and that of local segregation are the harmonic flow and the curl flow, respectively, and that a part of the small-world region is dominated by internal circulation of information flow.

## 1. Introduction

Recently, small-world topology of brain networks has been paid much attention in neuroscience. It is found ubiquitously in both structural and functional neuronal networks from those of local neuronal populations to large-scale brain areas (Bassett and Bullmore, 2006; Bullmore and Sporns, 2010; Poli et al., 2015). It has been suggested that the small-world topology is significant to brain functions because it balances integration and segregation of information processing on brain networks (Sporns and Zwi, 2004; Downes et al., 2012). Disruption of the small-world topology is suggested to be related to brain disease (Fornito and Bullmore, 2015).

Small-world topology is characterized by two structural metrics of networks. One is the mean path length and the other is the clustering coefficient. A network is called small-world when its mean path length is small and its clustering coefficient is large (Watts and Strogatz, 1998). Note that it is meaningful only for sparse networks since densely connected networks trivially satisfy the two features of the small-world topology (Markov et al., 2013).

A small value of the mean path length could make communication between any pair of nodes rapid and thus contribute to global integration of information. On the other hand, high clustering in a sparse network indicates that it consists of local groups of nodes that are densely connected within each group. This could support segregated specialized information processing. Thus, the small-world topology seems to compromise apparently opposite aspects of information processing on networks: integration and segregation. In other words, small-world networks can be characterized as both locally and globally efficient (Latora and Marchiori, 2001). However, what is relevant to functioning of a system is not structure but dynamical processes under the structural constraint (Barrat et al., 2008). The influence of the small-world topology on dynamical behaviors has been studied in the literature. For example, coexistence of fast response and coherent oscillations in the dynamics of networks of model neurons (Lago-Fernández et al., 2000) and improvement of synchronizability of general coupled identical oscillators (Barahona and Pecora, 2002) are achieved in the small-world regime of the Watts-Strogatz small-world network model (Watts and Strogatz, 1998). However, the quantitative effect of the small-world topology on information flow generated by dynamical processes on networks is still obscure.

In this paper, we do not concern whether the small-world topology is relevant to functioning of real-world brain networks. Rather, we take it for granted and study its influence on information flow generated by a dynamical process on the Watts-Strogatz small-world network model (Watts and Strogatz, 1998). We consider random threshold networks that have been used as a model of neural network dynamics (Kürten, 1988) for their simplicity and low computational costs (Rohlf, 2008). Information flow is quantified by the transfer entropy (Schreiber, 2000). For the analysis of information flow, we employ the combinatorial Hodge theory (Jiang et al., 2011). Miura and Aoki (2015a) used this technique to reveal global loop structure of an evolving neural network model and Miura and Aoki (2015b) showed that it can distinguish different learning rules. Fujiki and Haruna (2014) applied the combinatorial Hodge theory to study the influence of different degree distributions on the composition of information flow generated by a dynamical process on networks. The combinatorial Hodge theory enables us to decompose any flow on a network into three mutually orthogonal components: gradient, harmonic and curl flows. In succeeding sections, we study how the balance between these components in information flow changes as the parameter of the Watts-Strogatz model is varied by numerical simulation and discuss its implications.

## 2. Materials and methods

### 2.1. Random threshold networks on the small-world model

We employed the conventional *Watts-Strogatz model (WS model)* (Watts and Strogatz, 1998). It is constructed as follows. First, *N* nodes are arranged on a ring lattice and each node is connected to its 2*k* nearest neighbors (*k* < < *N*). For example, if *k* = 2, a node is connected to 4 other nodes: its two nearest neighbors and two second-nearest neighbors. Second, each edge is randomly rewired with probability *p* (0 ≤ *p* ≤ 1). *p* = 0 and *p* = 1 correspond to the lattice network and completely random networks (*Erdös-Rényi random networks*), respectively. For a certain range of *p* between these two extremes, we get so-called small-world networks with a small mean path length and a high clustering coefficient. In order to run random threshold networks on the WS model, we needed to assign a direction to each link. For each link, one of the two directions was chosen at random with equal probability. In this paper, we set *N* = 400 and consider the two cases *k* = 3 and *k* = 4. We also performed the same numerical simulation study except *N* = 200 and obtained the qualitatively similar results as those described below.

We simulated *random threshold networks (RTNs)* (Rohlf and Bornholdt, 2002) on the WS model. In RTNs, each node is assumed to take two states +1 and −1 corresponding to firing and resting states of a neuronal population, respectively. The state *x*_*i*_(*t*) of node *i* at time *t* is updated synchronously by the rule

(1) $$\begin{array}{l} x_{i}\left(t+1\right)=sgn\left(\overset{N}{\underset{j\;=\;1}{\sum }}w_{ij}x_{j}\left(t\right)+h_{i}\right), \end{array}$$

where *sgn*(*x*) = 1 if *x* ≥ 0 and *sgn*(*x*) = −1 otherwise. If there is a directed link from node *j* to *i*, we set *w*_*ij*_ = ±1 with equal probability. Otherwise, *w*_*ij*_ = 0. The threshold *h*_*i*_ for each node *i* is set to 0 in this paper.

The dynamics of RTNs can take three phases, ordered, critical and chaotic, depending on the values of the parameters (Kürten, 1988; Rohlf and Bornholdt, 2002; Rohlf, 2008; Szejka et al., 2008). For *N* = 400 and *k* = 3, 4, they exhibit weakly chaotic behaviors for all 0 ≤ *p* ≤ 1, namely, reside in the chaotic phase close to criticality, as we numerically verify below. These conditions were adopted in order to mimic spontaneous background activity of real-world neuronal networks (Chialvo, 2010).

In general, other things being equal, the dynamics tend to become unstable for larger values of *k*. To the best of our knowledge, no analytic condition for the boundary between the ordered and the chaotic phases for RTNs on the WS model is derived so far. However, the phase of RTNs can be numerically assessed by the behavior of damage spreading. In the chaotic phase, a damage applied to a node, namely, a flip of the state of the node, propagates indefinitely as the state of the system evolves and eventually a finite fraction of the whole nodes is influenced. On the other hand, the damage dies away in the ordered phase. At the critical phase, a flip propagates to exactly one succeeding node on average. The size of the influence can be quantified as follows (Gershenson, 2003). Let **x**(0) be a random initial state of an RTN on the WS model. A node is chosen at random and its state is flipped. Let **y**(0) be the resulting state of the whole system which is 1 bit away from **x**(0).

(2) $$\begin{array}{l} d\left(\text{x}\left(t\right),\text{y}\left(t\right)\right)=\frac{1}{N}\overset{N}{\underset{i\;=\;1}{\sum }}\frac{\left|x_{i}\left(t\right)-y_{i}\left(t\right)\right|}{2} \end{array}$$

is the Hamming distance between the two states after *t* time steps. Let

(3) $$\begin{array}{l} \delta_{t}=d\left(\text{x}\left(t\right),\text{y}\left(t\right)\right)-d\left(\text{x}\left(0\right),\text{y}\left(0\right)\right), \end{array}$$

where *d*(**x**(0), **y**(0)) = 1/*N* and $\delta^{*}=\underset{t\to \infty }{\lim}\delta_{t}$. δ^*^ > 0 indicates that the dynamic is sensitive to initial conditions and thus is an evidence for the chaotic phase. On the other hand, δ^*^ < 0 or δ^*^ = 0 mean that the dynamic is insensitive or neutral to perturbations and thus correspond to the ordered or the critical phases, respectively. Note that the asymptotic size of influence of perturbations is a well-known order parameter of Boolean network dynamics (Derrida and Pomeau, 1986). The quantity $\underset{t\to \infty }{\lim}d\left(\text{x}\left(t\right),\text{y}\left(t\right)\right)$ approximates it in a finite size system. Thus, the sign of δ^*^ is a convenient way to numerically assess the phase of Boolean network dynamics. This method has been applied to random Boolean networks on the WS model (Lizier et al., 2011) and here we have followed this approach.

**Figure 1:**
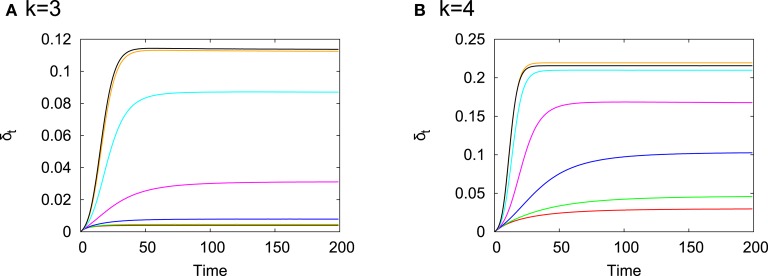
**Time evolution of δ_*t*_ for (A) *k* = 3 and (B) *k* = 4**. Each curve is the average over 100 random initial conditions for each realization of RTN, 100 realizations of RTNs on each network and 400 networks generated by the WS model with a specified value of *p*. *p* = 0.001000 (red), *p* = 0.003375 (green), *p* = 0.011391 (blue), *p* = 0.038443 (magenta), *p* = 0.129746 (cyan), *p* = 0.437894 (orange), and *p* = 0.985261 (black). These values of *p* were chosen so that they are arranged with an equal interval in the logarithmic scale because the small-world regime can be discriminated well in the logarithmic scale of *p* as we can see from Figure 5. Concretely, $p=p_{0}\times 1.5^{3n}$ for *p*_0_ = 0.001000 and 0 ≤ *n* ≤ 6.

Figure 1 shows time evolution of δ_*t*_ for *k* = 3 (a) and *k* = 4 (b). The rewiring probability *p* is varied within the range 10^−3^ ≤ *p* ≤ 1. For each *p*, δ_*t*_ > 0 in the depicted range of time and converges to a small positive value within a few tens to hundreds time steps. The system with *k* = 4 is more susceptible to perturbations than that with *k* = 3 and there is an overall tendency that the size of eventual damage influence becomes larger as *p* increases, namely, it becomes more remote from criticality.

### 2.2. Quantification of information flow

We quantified information transfer along each causal link in RTNs by the transfer entropy (Schreiber, 2000). Let us consider a directed link from node *j* to node *i*. The quantity

(4) $$\begin{array}{l} T_{j\to i}=H\left(X_{i}\left(t+1\right)|X_{i}\left(t\right)\right)-H\left(X_{i}\left(t+1\right)|X_{i}\left(t\right),X_{j}\left(t\right)\right) \end{array}$$

is a measure of information transfer from node *j* to node *i* and is called the *transfer entropy*. Here,

(5) $$\begin{array}{c} H\left(X_{i}\left(t+1\right)|X_{i}\left(t\right)\right)=-\underset{x_{i}\left(t\;+\;1\right),x_{i}\left(t\right)}{\sum }p\left(x_{i}\left(t+1\right),x_{i}\left(t\right)\right)\\ \;\times \log_{2}p\left(x_{i}\left(t+1\right)|x_{i}\left(t\right)\right) \end{array}$$

is the conditional entropy (Cover and Thomas, 1991) of the future state *x*_*i*_(*t* + 1) of node *i* given its present state *x*_*i*_(*t*) which represents the amount of average uncertainty to predict the *i*'s future state from its present state. *p*(*x*_*i*_(*t* + 1), *x*_*i*_(*t*)) is the joint probability that one observes a pair of states (*x*_*i*_(*t* + 1), *x*_*i*_(*t*)) and *p*(*x*_*i*_(*t* + 1)|*x*_*i*_(*t*)) is the conditional probability that the state of *i* at time *t* + 1 is *x*_*i*_(*t* + 1) given its state at time *t* is *x*_*i*_(*t*). On the other hand,

(6) $$\begin{array}{l} H\left(X_{i}\left(t+1\right)|X_{i}\left(t\right),X_{j}\left(t\right)\right)=-\underset{x_{i}\left(t\;+\;1\right),x_{i}\left(t\right),x_{j}\left(t\right)}{\sum }\\ p\left(x_{i}\left(t+1\right),x_{i}\left(t\right),x_{j}\left(t\right)\right)\;\times \log_{2}p\left(x_{i}\left(t+1\right)|x_{i}\left(t\right),x_{j}\left(t\right)\right). \end{array}$$

is the conditional entropy of the future state of node *i* given its present state and *j*'s present state. The joint and conditional probabilities involved in Equation (6) are defined similarly as in Equation (5). Thus, the transfer entropy *T*_*j*→*i*_ (Equation 4) can be interpreted as the reduction of average uncertainty when one incorporates the knowledge about *j*'s present state into the prediction of *i*'s future state from its own present state.

In this paper, *T*_*j*→*i*_ on a fixed network was numerically estimated as follows. First, a network was generated from the WS model with given parameter values and fixed. Second, for each realization of RTN on the fixed network, 1000 time steps after disregarding initial 100 transient steps from a random initial condition were used to calculate *T*_*j*→*i*_. Finally, *T*_*j*→*i*_ was averaged over 100 realizations of RTNs on the fixed network. The average of *T*_*j*→*i*_ is also denoted by *T*_*j*→*i*_ by abuse of notation. The length of transient time steps was determined from the inspection of Figure 1. δ_*t*_s almost converge after 100 time steps for all *p*. This indicates that we can regard that the dynamics of RTNs settle down to the stationary regime after 100 time steps and the probability distributions involved in the formula of *T*_*j*→*i*_ are well-defined.

Let us introduce a quantity *e*_*ij*_ as follows. *e*_*ij*_ = *T*_*i*→*j*_ if there is a directed link from *i* to *j*, *e*_*ij*_ = −*T*_*j*→*i*_ if there is a directed link from *j* to *i* and *e*_*ij*_ = 0 if there is no link between *i* and *j*. *e*_*ij*_ defines a skew-symmetric matrix *e* = (*e*_*ij*_). Namely, *e* satisfies *e*_*ij*_ = −*e*_*ji*_ for all 1 ≤ *i, j* ≤ *N*. We call *e information flow*. Here, we have defined information flow as such because the combinatorial Hodge decomposition can be applied to only skew-symmetric matrices. (In general, if one has a matrix representing correlation, coupling strength, etc. between nodes, first one can decompose it into the sum of the symmetric part and the skew-symmetric part and then apply the Hodge decomposition to the latter.)

In the literature, information theoretic quantities such as transfer entropy are sometimes used for the purpose of causality detection (Hlaváčková-Schindler et al., 2007). In this paper, causality between two nodes is taken for granted. *T*_*j*→*i*_ quantifies the magnitude of impact of *j* on *i* along the causal relationship when there is a directed link from *j* to *i*. We emphasize that by definition *e*_*ij*_ = 0 for pairs (*i, j*) that are not connected. Although *T*_*j*→*i*_ could be positive for such pairs, they are ignored because we here conceive information flow as influence of one node to the other node along a causal link between them.

### 2.3. Combinatorial hodge decomposition

An *edge flow* on a network of size *N* is an *N* × *N* skew-symmetric matrix *e* = (*e*_*ij*_) satisfying *e*_*ij*_ = 0 for all pairs of nodes (*i, j*) that are not connected. The information flow introduced in the last subsection is an instance of edge flow.

An edge flow *e* = (*e*_*ij*_) can be uniquely decomposed into three orthogonal components via the combinatorial Hodge decomposition theorem (Jiang et al., 2011): gradient *g* = (*g*_*ij*_), harmonic *h* = (*h*_*ij*_) and curl *c* = (*c*_*ij*_) flows. A *gradient flow g* is an edge flow that can be written as the difference of a potential function. Namely, there exists a real-valued function *f* on the set of nodes such that *g*_*ij*_ = *f*_*j*_ − *f*_*i*_ for all pairs (*i, j*) that are connected. A *harmonic flow h* is a non-gradient edge flow that is also curl-free. Namely, *h* vanishes on every triangle {*i, j, k*} (any pair of nodes from {*i, j, k*} is linked) in the sense that *h*_*ij*_ + *h*_*jk*_ + *h*_*ki*_ = 0. A *curl flow c* is defined by *c* = *e* − *g* − *h* and thus is non-gradient and may have non-zero curls on some triangles. One can say that the harmonic flow represents the globally circulating component of a given edge flow, while the curl flow corresponds to the local circulating one. We call the sum of the harmonic and the curl flows *loop flow* and denote it by *l* = *h* + *c*. Note that *l* represents the non-gradient flow and is precisely equal to the divergence-free flow which is a result of elementary linear algebra. Here, the divergence of an edge flow *e* at node *i* is given by the sum of *e*_*ij*_ over all *j* connected to *i*. If the divergence of *e* is zero at a node, the flow is conserved at the node, namely, the sum of incoming flows and the sum of outgoing flows are equal. It follows that vanishing of the divergence of nonzero *e* at every node implies that it contains a loop along which each element of *e* is positive.

The magnitude of an edge flow *e* can be measured by its *l*^2^-norm $||e|{|}^{2}=\underset{i,j}{\sum }e_{ij}^{2}$. Adopting the *l*^2^-norm has a certain advantage since we have the equality

(7) $$\begin{array}{l} ||e|{|}^{2}=||g|{|}^{2}+||l|{|}^{2}=||g|{|}^{2}+||h|{|}^{2}+||c|{|}^{2} \end{array}$$

due to the orthogonality of the decomposition. We can define the relative strength of each component by γ = ||*g*||^2^/||*e*||^2^, η = ||*h*||^2^/||*e*||^2^ and χ = ||*c*||^2^/||*e*||^2^ called *gradient ratio, harmonic ratio* and *curl ratio*, respectively (Fujiki and Haruna, 2014). The sum of them is 1 by Equation (7). We also introduce λ = ||*l*||^2^/||*e*||^2^ and call it *loop ratio*.

Each component constitutes a linear subspace of the finite-dimensional vector space consisting of all edge flows. Thus, it makes sense that we talk about the dimension of the subspace consisting of all gradient flows, and so on. We define the relative size of each subspace by the ratio of the dimension of the subspace to the dimension of the space of all edge flows. Let Γ be the relative size of the subspace of gradient flows, *H* that of harmonic flows, *X* that of curl flows and Λ that of loop flows. We call them *structural gradient ratio, structural harmonic ratio, structural curl ratio* and *structural loop ratio*, respectively. Note that these structural ratios are determined by the underlying network alone, while ratios denoted by lower-case Greek letters defined above depend on each edge flow. In particular, the latter quantities are a function of dynamical processes on the network for information flows. Note also that each structural ratio is equal to the average relative strength of corresponding component of edge flows of a fixed *l*^2^-norm chosen uniformly at random. Thus, we can quantitatively evaluate whether a dynamical process on a given network enhance or diminish intrinsic strength of each component determined by network topology alone by comparing the relative strength of that component for the information flow generated by the dynamical process to the corresponding structural ratio.

The gradient and curl components of a given edge flow can be numerically computed by solving corresponding least square optimization problems. Here, we obtained them by computing the Moore-Penrose inverses of appropriate matrices (Jiang et al., 2011). The computation of curl components involves manipulation of matrices whose size is the number of triangles. This requires high computational costs when the underlying network is close to the lattice network. This is the reason why we restricted our numerical simulations to networks with modest sizes (*N* ≤ 400).

**Figure 2:**
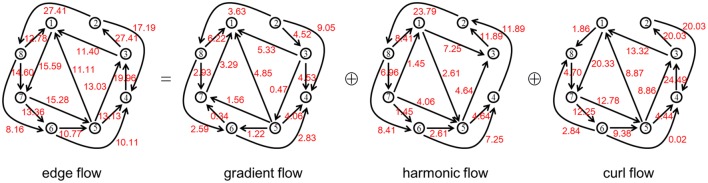
**An example of edge flow on a network generated from the WS model with *N* = 8, *k* = 2 and *p* = 0.1 and its combinatorial Hodge decomposition into the three components**. The value of each flow is rounded off to the four decimal places and multiplied by 10^2^ for visibility.

Figure 2 illustrates the combinatorial Hodge decomposition of an information flow *e* obtained by the procedure described in Section 2.2 on a network generated from the WS model with *N* = 8, *k* = 2 and *p* = 0.1. For this example, we have ||*e*||^2^ = 0.411, ||*g*||^2^ = 0.028, ||*h*||^2^ = 0.122, ||*c*||^2^ = 0.261 and ||*l*||^2^ = 0.383. Thus, the relative strength of each component is: γ = 0.069, η = 0.297, χ = 0.634 and λ = 0.931. On the other hand, the structural ratios are: Γ = 7/16 = 0.4375, *H* = 1/16 = 0.0625, *X* = 8/16 = 0.5 and Λ = 9/16 = 0.5625.

## 3. Results

The information flow generated by RTNs on the WS model was decomposed into the three components. In this section, all quantities are averaged over 400 networks for each parameter set of the WS model and error bars in figures represent the standard deviations.

**Figure 3:**
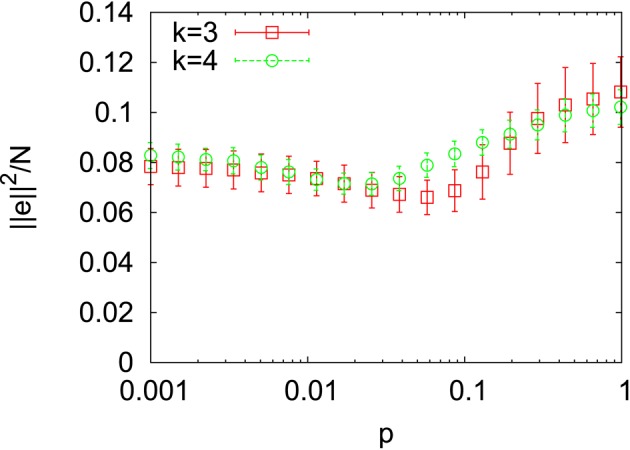
**The magnitude of information flow divided by the number of nodes *N* = 400 for *k* = 3 and *k* = 4**.

The magnitude of information flow ||*e*||^2^ divided by the number of nodes *N* is shown in Figure 3 for *k* = 3 and *k* = 4. The range of this quantity is confined within an interval well apart from the zero in both cases. This indicates that non-trivial information flows were generated for all values of *p*. One might expect that the magnitude of information flow becomes large as the randomness of the underlying network is strengthened since we have observed that the dynamic becomes more unstable as *p* increases in Figure 1. However, we can see a minimum of ||*e*||^2^/*N* for both *k* = 3 and *k* = 4 in Figure 3. It could result from the small-world topology since the minimum points are contained in the small-world region as we define below (see **Figure 5**). However, the exact reason for this unexpected non-linear behavior is obscure at present. In the following, we concentrate on the relative strength of components of information flow and leave it as future work.

**Figure 4:**
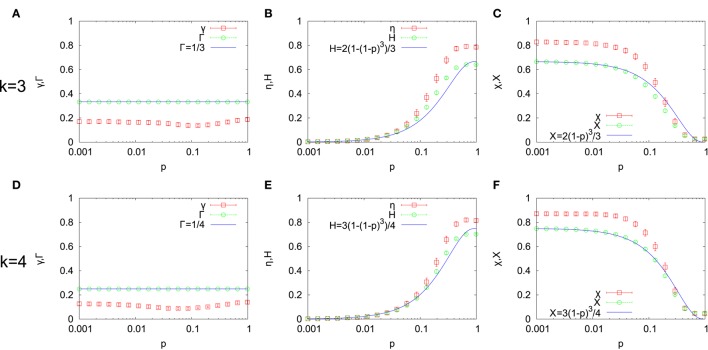
**The relative strength of gradient γ (A,D), harmonic η (B,E) and curl χ (C,F) flows together with corresponding structural ratios are shown for *k* = 3 (top row) and *k* = 4 (bottom row)**.

In Figure 4, the relative strength of each component of information flow is shown together with the corresponding structural ratio. The gradient ratio γ is significantly smaller than the structural gradient ratio Γ for all *p* in both *k* = 3 (Figure 4A) and *k* = 4 (Figure 4D). This indicates that information flows generated by RTNs favor the loop component. The value of Γ can be theoretically obtained. Indeed, the dimension of the space of edge flows is just the number of edges and is equal to *kN*. The dimension of the subspace of gradient flows is the number of nodes minus the number of connected components of the underlying network. However, the latter can be assumed to be negligible compared to *N* in the setting of our numerical simulations. Thus, Γ = *N*/(*kN*) + *O*(1/*N*) ≈ 1/*k* which does not dependent on *p*. This agrees well with the result of numerical simulations as shown in Figures 4A,D.

Figures 4B,E show the harmonic ratio η and the structural harmonic ratio *H*. Both increase as *p* increases, namely, the underlying network becomes more random. When *p* is close to 1, η is significantly larger than *H*. On the other hand, the curl ratio χ and the structural curl ratio *X* decrease as *p* increases. For small values of *p*, χ is significantly larger than *X* as shown in Figures 4C,F. Thus, the dominant part of the loop component is enhanced by information flow generated by RTNs. Namely, when the network is close to the lattice network, the curl component is enhanced while the harmonic component is enhanced for networks close to Erdös-Rényi random networks. We can give simple theoretical estimations of *X* and *H*. Let us first consider the lattice network (*p* = 0). In this case, *H* = 0 since any loop of length greater than 3 can be expressed as a “sum” of triangles. Hence, the dimension of the subspace of curl flows is the dimension of the space of edge flows (*kN*) minus the dimension of the subspace of gradient flows (*N* − 1). Thus, *X* = (*kN* − (*N* − 1))/*kN* = (*k* − 1)/*k* + *O*(1/*N*) for *p* = 0. Now, let us assume *p* > 0. The dimension of the subspace of curl flows is the number of “linearly independent” triangles. By the random rewiring process, these triangles in the lattice network may be destroyed. The number of triangle decreases with multiplication factor (1 − *p*)^3^ up to *O*(1/*N*) terms in the WS model (Barrat and Weigt, 2000). If we assume that the number of “linearly independent” triangles linearly scales with the number of triangles, then we predict *X* = (*k* − 1)(1 − *p*)^3^/*k*+*O*(1/*N*) for *p* > 0. For *H*, we have *H* = 1 − Γ − *X* = (*k* − 1)(1 − (1 − *p*)^3^)/*k* + *O*(1/*N*). These predictions agree well at least for small *p* > 0 as we can see from Figures 4B,C,E,F. The *O*(1/*N*) correction can be estimated for *p* = 1 which is visible in the scale of Figure 4. When *p* = 1, the expected number of triangles is 4*k*^3^/3 (Newman, 2010). Thus, our prediction is *X* = 4*k*^2^/(3*N*) for *p* = 1. Since *N* = 400, we have *X* = 0.03 and *X* = 0.0533⋯ for *k* = 3 and *k* = 4, respectively.

**Figure 5:**
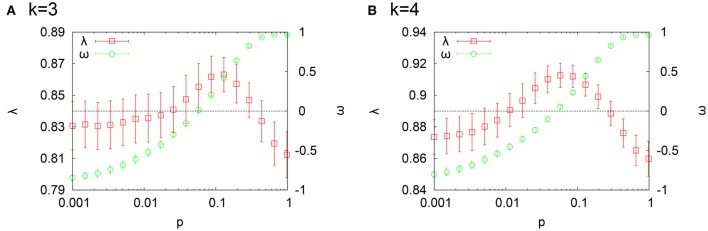
**The loop ratio λ is compared with a small-world index ω**. **(A)** *k* = 3 and **(B)** *k* = 4.

One can be aware of a small hollow in Figures 4A,D at an intermediate value of *p*. Its counterpart λ(= 1 − γ = η + χ) is enlarged in Figure 5. We also show a small-world index ω (Telesford et al., 2011) together. Another small-world index was suggested by Humphries and Gurney (2008) earlier. Here, we adopted the former because it better discriminates the small-world region. For a given network generated by the WS model, it is defined by ω = *L*_*r*_/*L* − *C*/*C*_*c*_, where *L* is the mean path length of the network, *L*_*r*_ is the average of the mean path length of Erdös-Rényi networks with the same numbers of nodes and links (*p* = 1), *C* is the clustering coefficient of the network and *C*_*c*_ is the clustering coefficient of the lattice network with the same *k* and *N* (*p* = 0). ω has a value within the range −1 < ω < 1 and the network is judged to be small-world if ω is close to 0. As ω varies toward −1, the network is more like a lattice network. On the other hand, the network becomes more like a random network as ω approaches 1. From Figure 5, we can see that the loop ratio λ takes its maximum value within the small-world region (If one would like to decide the boundary, one could take −0.5 ≤ ω ≤ 0.5 as the small-world region as suggested by Telesford et al., 2011). When *k* = 3 (Figure 5A), the value of *p* such that λ is maximum slightly shifts toward *p* = 1 from *p* satisfying ω = 0. We also obtained the similar shift toward *p* = 1 for both *k* = 3, 4 when *N* = 200 but still the maximum point of λ is contained in the small-world region (data not shown).

## 4. Discussion

The aim of this paper is to reveal the influence of the small-world topology on information flow generated by dynamical processes on it. In this paper, we studied the composition of information flow generated by RTNs on the Watts-Strogatz small-world model. Information flows were decomposed into three mutually orthogonal components by the combinatorial Hodge theory: gradient, harmonic and curl flows. The result for the structural ratios showed that networks close to the lattice network have a larger capacity to support locally circulating curl flows while those close to the Erdös-Rényi random networks favor globally circulating harmonic flows. The result for the relative strengths of harmonic and curl flows indicated that the dominant component of loop flows at fixed *p* is enhanced in information flows generated by RTNs (Figure 4). Furthermore, the relative strength of loop flows which is the sum of those for harmonic and curl flows takes its maximum value in the small-world region (Figure 5). This result suggests that the small-world topology promotes circulating information transfer generated by dynamical processes on it.

In the literature, the small-world topology has often been associated with a balance between integration and segregation of information processing (Sporns and Zwi, 2004; Downes et al., 2012). In terms of this point of view, our result in this paper can be interpreted as follows. Harmonic flow represents the globally circulating component of information flow and thus related to global integration of information processing. On the other hand, curl flow represents the locally circulating component of information flow and thus related to local segregation of information processing. The sum of relative strengths of them λ takes its maximum value within the small-world region. This result can be seen as a representation of a balance between integration and segregation of information processing achieved in the small-world region. Note that the maximum point of λ tends to shift toward more random side within the small-world region. Although our result is based on synthetic data, it could shed a new light on the interpretation of the result that several real-world brain networks reside more random part of the small-world region far away from the maximally small-world point (Muller et al., 2014). Anyway, taking dynamical processes on networks into account is important to assess functions supported by the network topology.

The influence of the small-world topology on performance of artificial neural networks has been studied so far. Kim (2004) and Oshima and Odagaki (2007) showed that memory capacity of the Hopfield neural network is enhanced as the network becomes more random in the WS model. However, the neural network of the nematode *Caenorhabditis elegans* (Varshney et al., 2011) is organized as small-world and has lower memory capacity than that of fully random networks (Kim, 2004; Oshima and Odagaki, 2007). What is the reason for the fact that natural selection does not select network topologies with an optimal performance? They discussed that one factor is the wiring cost to make long spatial connections which was not considered in their numerical experiments. The reason why high clustering diminishes memory capacity is still obscure. However, memory capacity is just one of many functions of brain networks. In particular, it is a global function of a network since patterns are stored as distributed synaptic strengths within the whole network. In contrast, our analysis took into account both global and local functions although less concrete. We identified not only the positive influence of small mean path length on the composition of information flow but also that of high clustering as shown in Figure 4.

In conclusion, our approach in this paper using the combinatorial Hodge theory provides a new tool to analyze information flow generated by dynamical processes on networks. In addition to apply this method to studying effects of various network structures such as degree correlations, network motifs and community structure in mathematical models, applications to real-world multivariate time series data that are becoming available by progress in multi-site recording techniques are of importance for future work to further assess the limit and applicability of this approach.

## Author contributions

TH and YF designed and performed research. TH wrote the paper.

## Funding

This work was partially supported by JSPS KAKENHI Grant Number 25280091.

### Conflict of interest statement

The authors declare that the research was conducted in the absence of any commercial or financial relationships that could be construed as a potential conflict of interest.
